# Economic burden of Chagas disease in Brazil: a nationwide cost-of-illness study

**DOI:** 10.1016/j.lana.2025.101202

**Published:** 2025-08-08

**Authors:** Mônica Viegas Andrade, Kenya Valeria Micaela de Souza Noronha, Aline de Souza, Nayara Abreu Julião, André Soares Motta-Santos, Paulo Estevão Franco Braga, Henrique Bracarense, Yasmim Caroline Silva, Bruno Ramos Nascimento, Mariângela Carneiro, Francisco Rogerlândio Martins-Melo, Isis Eloah Machado, Pablo Perel, Yvonne Geissbühler, Caroline Demacq, Antônio Luiz Pinho Ribeiro

**Affiliations:** a Economics Department, Center for Development and Regional Planning, Universidade Federal de Minas Gerais, Belo Horizonte, Brazil; b Center for Health Technology Assessment, Universidade Federal de Minas Gerais, Belo Horizonte, Brazil; c Department of Internal Medicine, Faculty of Medicine, Universidade Federal de Minas Gerais, Belo Horizonte, Brazil; d Telehealth Center and Cardiology Service, Hospital das Clínicas, Universidade Federal de Minas Gerais, Belo Horizonte, Brazil; e Department of Parasitology, Universidade Federal de Minas Gerais, Belo Horizonte, Brazil; f Federal Institute of Education, Science and Technology of Ceará, Fortaleza, Brazil; g Department of Family Medicine, Mental and Collective Health, Universidade Federal de Ouro Preto, Belo Horizonte, Brazil; h World Heart Federation, Geneva, Switzerland; i Novartis Global Health, Basel, Switzerland

**Keywords:** Chronic Chagas disease, Economic burden, Societal perspective, Direct medical costs, Indirect costs, Absenteeism, Markov model, Brazil

## Abstract

**Background:**

Chagas disease remains a public health issue with substantial financial impact on the healthcare system of Latin American countries. Despite its great economic burden, research quantifying the direct and indirect costs are limited, particularly within Brazil. This study estimates the economic burden of chronic Chagas disease in Brazil, as part of the broader project, ‘The Burden of Chagas Disease in the Contemporary World: The RAISE Study’.

**Methods:**

A Markov model was used to estimate the economic burden of chronic Chagas disease from a societal perspective considering six mutually exclusive health states: four clinical forms (indeterminate, cardiac, digestive, mixed) and two absorptive states (death and cure). This model was analyzed through microsimulation with a one-year cycle length, considering a hypothetical cohort of 10,000 patients, each repeated 1000 times to report the average. Data on costs were gathered, converted to 2024 purchasing power parity US dollars, and considered direct medical costs and productivity losses due to absenteeism.

**Findings:**

The annual economic burden of chronic Chagas disease in Brazil was estimated at $11.44 billion, constituting 0.23% of the gross domestic product, with a lifetime cost per patient of $45,034. Lifetime direct medical costs represent around 72% of the total lifetime economic burden, while indirect costs, 28%. Annual direct medical costs represent around 11% of the Ministry of Health budget.

**Interpretation:**

The significant economic burden highlights the necessity for effective public health policies and resource allocation in Brazil's healthcare system. Given the universal health coverage model, understanding these costs can guide improvements and interventions aimed at reducing Chagas disease’s impact.

**Funding:**

Funding was provided by Novartis Pharma AG as part of a research collaboration with the 10.13039/501100015708World Heart Federation, project number CLCZ696D2010R.

**Research in context**

**Evidence before this study**

A systematic review was undertaken to synthesize the literature concerning the costs associated with the treatment of Chagas disease on a global scale, encompassing both direct and indirect costs. Comprehensive electronic searches were performed in the Medline (via PubMed), Lilacs (via BVS), and Embase databases and included references published up to May 31, 2022. Relevant descriptors such as “Costs and Cost Analysis,” “Economics,” “Cost Allocation,” “Health Care Costs,” “Chagas Disease,” “American Trypanosomiasis,” and other synonyms and alternative terms were employed. The review imposed no language or location restrictions. Both complete and partial economic analyses were considered for inclusion.

A total of fifteen studies were selected, with approximately two-thirds of them assessing the economic burden in endemic countries. Adopting the same selection criteria as in our original systematic review, the electronic search was updated as of June 1, 2025, and three other publications were found. Considering the eighteen studies, the cost components most frequently analyzed included inpatient care, diagnostic examinations, surgical interventions, consultations, pharmaceuticals, and pacemakers. There was significant heterogeneity in the methodologies employed for cost estimation and data presentation, indicating a lack of standardization in the measurement techniques and cost components. Hospitalization emerged as the most frequently assessed component utilizing a consistent metric. The mean annual hospital cost per patient, expressed in 2024 purchasing power parity US dollars (PPP-USD), varied widely, ranging from $27.00 to $19,957.98. Additionally, the lifetime hospital cost per patient exhibited variability, ranging from $222.06 for general care to $15,216.45 for patients diagnosed with heart failure. Only one study estimated global economic burden considering 33 countries with prevalence of Chagas disease. Studies estimating the economic costs of Chagas disease in Brazil are quite limited and focused on specific institutions.

**Added value of this study**

This study seeks to address the gap in understanding Chagas disease economic burden in Brazil by employing a societal perspective and a Markov model to estimate the lifetime direct and indirect costs associated with chronic Chagas disease. It forms part of a broader project, “The Burden of Chagas Disease in the Contemporary World: The RAISE Study”. Incorporating up-to-date, country-specific cost parameters, this research provides a comprehensive analysis relevant to policymakers and healthcare planners. The principal findings reveal that the annual economic burden associated with chronic Chagas disease is approximately $11.44 billion, representing a significant portion of Brazil's Gross Domestic Product at 0.23%. Focusing specifically on direct medical expenses, these costs constitute 11% of the Ministry of Health's budget and 1.92% of total health expenditures. As Brazilian Unified Health System (SUS) offers universal and comprehensive healthcare coverage, this study highlights the importance of financial protection and access to healthcare resources for affected individuals and families.

**Implications of all the available evidence**

The findings underscore the critical need for targeted public health policies and interventions aimed at Chagas disease management, especially in the context of Universal Health Coverage debate. Addressing the economic burden of neglected chronic diseases, such as Chagas disease, provides valuable insights into strategies for efficient health resource allocation, particularly in middle- and low-income countries facing a dual challenge of managing endemic diseases and rising healthcare costs associated with chronic conditions.

## Introduction

Chagas disease is a neglected tropical disease caused by the protozoan *Trypanosoma cruzi*, primarily transmitted to humans by contact with contaminated feces/urine of blood-sucking triatomine bugs. Other transmission routes include blood transfusion, organ transplantation, congenital, and food-borne transmission.^1^^,^^2^ The disease progresses through two distinct phases: the acute, which is usually asymptomatic or may present as a febrile syndrome, without specific findings, and the chronic, which can lead to severe conditions such as Chagas chronic cardiomyopathy and megaesophagus.

Chagas disease remains a public health issue in 21 Latin American countries, affecting about 6 million people and causing 10,000–14,000 deaths annually.^1^ Due to increased migration in last decades, Chagas disease has become a condition of increasing global presence, with infections being detected in several non-endemic countries.^1^^,^^3^ Brazil accounts for approximately 3.7 million cases and recorded the highest number of Chagas disease-related deaths from 1990 to 2019.^4^, ^5^, ^6^ Currently, the high prevalence of chronic Chagas disease, resulting from infections acquired in past decades, imposes substantial direct and indirect costs to the healthcare system and families, driven by the long-term severity of disease complications.^4^^,^^7^ Direct costs include medical consultations, diagnostic tests, medications, prolonged hospitalizations, and complex procedures such as device implantation and transplant for patients with Chagas chronic cardiomyopathy. Indirect costs encompass productivity losses associated with absenteeism and presenteeism, premature mortality, and intangible costs reflecting the impaired quality of life experienced by Chagas disease patients.^5^^,^^7^^,^^8^

Despite its considerable significance, the economic burden of Chagas disease remains underexplored. To date, only three studies have been published: one with a global scope^9^ and two focusing on specific Latin American Countries.^8^^,^^10^ The global study, including 33 countries that reported Chagas disease cases over the past 15 years, estimated the disease's annual costs at USD 627.46 million (in 2012 values) and a burden of 806,170 DALYs per year. Brazil had the highest annual costs in Latin American Countries, USD 129.21 million, with the most considerable DALY burden and nearly one-quarter of the global morbidity.^9^ Country-specific studies have been conducted for Colombia^8^ and Ecuador,^10^ estimating annual costs at USD 13.1 (2017) and USD 37.5 (2003) million, respectively.

The limited research on economic burden of Chagas disease in Brazil represents a critical gap, given the country's prominent role in the global epidemiological landscape. Although Lee et al.’s^9^ global study includes Brazil, the country is grouped with other middle- and low-income nations, with estimates predominantly resulting from extrapolation of primary data from Argentina and Colombia. These estimations may not accurately capture Brazil's unique expenditure patterns. Understanding the economic burden of Chagas disease in Brazil is crucial given the country's central role in the fight against the disease and its importance on the Universal Health Coverage debate. Brazil has the Unified Health System (SUS, *Sistema Único de Saúde*) that provides a free of charge and comprehensive healthcare coverage, including high-complexity procedures. This context is particularly relevant for estimating the economic burden of diseases, as SUS provides financial protection, particularly for families that rely exclusively on its services, without access to private insurances. Finally, the country leads global initiatives like those of Unitaid, investing in new tools for diagnosis and prevention, with a particular focus on maternal-fetal transmission.^11^^,^^12^ These factors underscore the importance of investigating the economic burden of Chagas disease in Brazil to support effective public policies and strengthen global efforts against the disease. This study aims to address this gap by estimating the economic burden of chronic Chagas disease in Brazil. It forms part of a broader project, “The Burden of Chagas Disease in the Contemporary World: The RAISE Study”. Notably, this is the first analysis of the economic burden of chronic Chagas disease in the country that adopts a societal perspective and incorporates country-specific and up-to-date cost parameters.

## Methods

### Study overview

In this nationwide cost-of-illness study, chronic Chagas disease economic burden in Brazil was assessed from the societal perspective using Markov model approach built in TreeAge® Pro 2009 (https://www.treeage.com/). Expenditure data were obtained at current prices in Brazilian Real (R$) and converted into 2024 purchasing power parity US dollars (2024 PPP-USD) using a cost converter tool.^13^ This conversion was performed to ensure international comparability by adjusting for differences in the cost of living among countries. The study was approved by the Research Ethics Committee of the Federal University of Minas Gerais (Protocol number 74852723.4.0000.5149) and was conducted in accordance with the Consolidated Health Economic Evaluation Reporting Standards (CHEERS) guidelines (Supplementary Table S1).

### Markov model

**Fig. 1:**
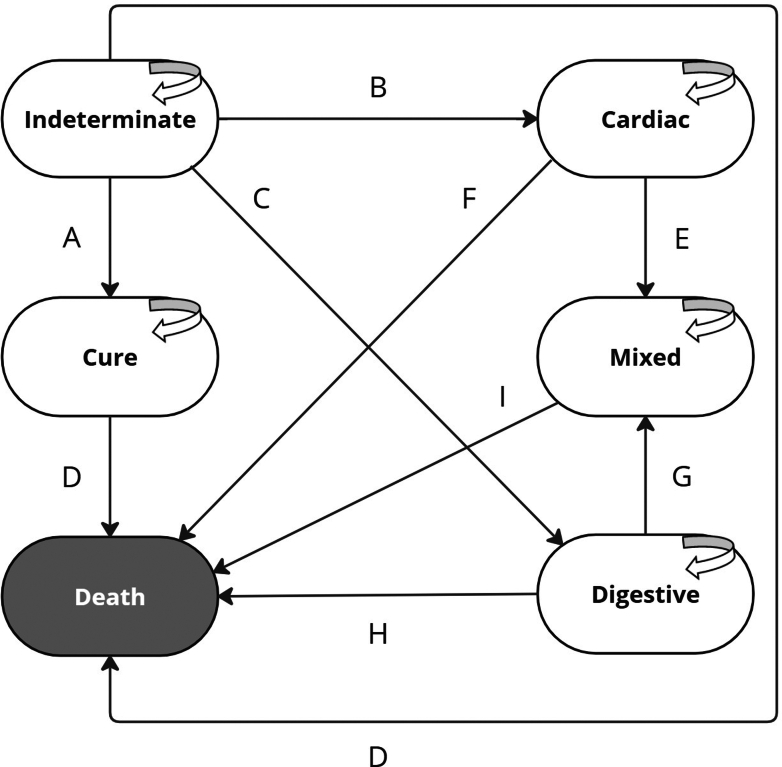
**Individual-based chronic Chagas disease Markov model structure.** Notes: **A** Transition probability from indeterminate form to cure (8.0%). **B** Transition probability from indeterminate form to cardiac form (3.0%). **C** Transition probability from indeterminate form to digestive form (1.1%). **D** Transition probability from indeterminate form to death or from de cure to death (general mortality rate for population over 14 years old). **E** Transition probability from cardiac form to mixed form (1.1%). **F** Transition probability from cardiac form to death (7.9%). **G** Transition probability from digestive form to mixed form (3.0%). **H** Transition probability from digestive form to death (0.065% + general mortality rate for population over 14 years old). **I** Transition probability from mixed form to death (7.9%).

Markov models are helpful in estimating costs and health outcomes of chronic diseases over the individuals’ lifetime. Each individual can follow a specific prognosis formed by transition probabilities of different paths.^14^ In this study, the Markov model includes six mutually exclusive health states defined by the natural history of the disease: four clinical forms of chronic Chagas disease (indeterminate, cardiac, digestive, and mixed) and two absorptive states (death and cure) (Fig. 1). This model was analyzed through microsimulation with a one-year cycle length, considering a hypothetical cohort of 10,000 patients, each repeated 1000 times to report the average.

At the beginning of the Markov model, individuals were distributed among health states based on recent meta-analysis on the prevalence of chronic Chagas disease clinical forms^15^: 47.2% allocated to the indeterminate form, 25.7% to the cardiac, 19.5% to the digestive, and 7.7% to the mixed form (cardiac + digestive). Due to the lack of specific data, and to the difficulties to define it as a separate clinical entity rather than sequelae, the thromboembolic form, including stroke, was not separately analyzed as a clinical form. Individuals in the indeterminate form could only be cured during the first cycle and were not susceptible to reinfection. As cure is not possible from the cardiac and digestive forms, individuals could transit to the mixed form, death, or remain in their respective health conditions. The time horizon was 65 years because by the end of the 65 cycles nearly all individuals were expected to have died.

**Fig. 2:**
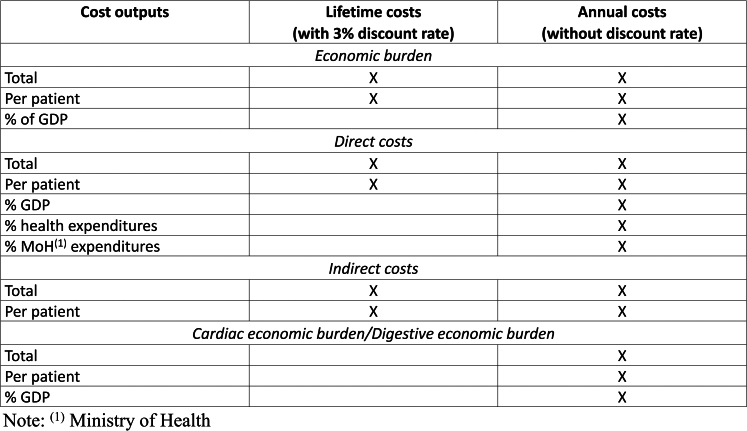
**Cost outputs estimated for chronic Chagas disease in Brazil**.

The economic burden of chronic Chagas disease comprised direct medical costs and indirect costs due to absenteeism. The cost outputs include lifetime and annual costs, evaluated both in total and per patient. Annual costs were further assessed as the percentage of total health expenditures, Ministry of Health expenditures and Gross Domestic Product (GDP). Additionally, total and per-patient annual costs were separately analyzed for the cardiac and digestive forms of the disease by distributing all individuals in each respective form at the beginning of Markov model (Fig. 2).

A 3% discount rate was applied to lifetime costs, aligned with practices in several countries and recent guidelines.^16^ Annual costs per patient were calculated by dividing the average lifetime costs (without discounting) by the expected years of life in the Markov model. Total costs were calculated by multiplying per-patient costs by the estimated prevalence of Chagas disease in Brazil. To account for the declining incidence of Chagas disease over the past two decades, prevalence data from 2010, estimated at 2.4%,^17^ were used for individuals aged 15 years and older.

Health outputs included quality-adjusted life years (QALYs), years of life lost, and health-related quality of life (HRQoL) loss. QALYs were estimated using the baseline Markov model that includes quality-adjusted life factors calculated as one minus the disability weights for cardiac and digestive sequelae provided by the Institute for Health Metrics and Evaluation (IHME).^18^ For the cardiac form, the disability weights correspond to patients with heart failure and atrial fibrillation. Proportions of patients within these conditions were obtained from the SaMi-Trop Cohort study^19^ (Supplementary Table S2). Years of life lost were calculated by comparing QALYs from the baseline model, that uses Chagas disease-specific mortality rates, with those from a model applying general population mortality rates. To compute HRQoL loss, the baseline model was compared to a specification that excluded disability weights. A separate analysis was also conducted for cardiac and digestive forms. A discount rate was not applied in estimating health outputs to accurately quantify the number of life years individuals are expected to lose due to chronic Chagas disease.

### Transition probabilities

The annual transition probabilities between different states, including death from cardiac and from digestive forms, were derived from multiple sources and validated by a panel of experts (Supplementary Table S3). For individuals in the indeterminate form, the yearly likelihood of mortality was assumed equal to that of the general population. Official mortality data for the Brazilian population aged 15 years and older were used.^20^ These rates were adjusted on an annual basis to account for age progression over each cycle (Supplementary Table S4). The probability of mortality from the mixed form was determined by selecting the higher value between the cardiac and digestive forms.

### Direct costs

Multiple databases were utilized to estimate the average annual direct costs associated with each health state. These costs included expenditures on medications, outpatient care, and inpatient services.

#### Outpatient care and medication

The package and frequency of outpatient services and medications were based on four published clinical protocols,^1^^,^^4^^,^^21^^,^^22^ validated by a panel of specialists (Supplementary Tables S5 and S6). Outpatient costs were obtained from an official public database of reimbursements to private healthcare providers in Brazil.^23^ The annual number of physician visits and the likelihood of consulting a physician were derived from the 2019 National Health Survey (PNS - *Pesquisa Nacional de Saúde*),^24^ which considered data for individuals who reported having been diagnosed with several chronic diseases, including Chagas disease. However, the survey does not differentiate between Chagas disease clinical forms. For individuals in the indeterminate form, the probability of visiting a doctor was based on data reported by the general population. For cardiac form, probabilities were derived from individuals with Chagas disease who also reported cardiac complications, while for the digestive form, solely from individuals diagnosed with Chagas disease.

Medicine costs were estimated considering the posology to a reference individual weighting 70 kg (Supplementary Table S6). Price of medicines were obtained from the official website managed by the Brazilian Ministry of Health.^25^

#### Inpatient care

The average cost per hospitalization was calculated using data from the Diagnosis Related Group Brazil (DRG-Brasil), a closed database organized by a private company that audits and classifies hospitalizations according to DRG coding system.^26^ The sample includes public and private hospitals that hire this company to improve their performance. Data from 2022 to 2023 were requested. Hospitalizations that had primary and secondary International Classification of Disease (ICD-10) codes related to Chagas disease forms were selected and categorized into two groups: clinical and surgical. Subsequently, the average cost was estimated for each category and form considering the frequency of patients undergoing each procedure (Supplementary Table S7). Average costs for the mixed form were defined by the higher value between cardiac and digestive forms. For heart transplants, average cost was estimated using data from the Hospital Admission Information System^27^ of the public healthcare system as virtually all heart transplants in Brazil are funded by SUS.

Information regarding the likelihood of hospitalization on an annual basis was obtained through 2019 PNS, specifically considering individuals who reported Chagas disease and heart disease (Chagas Cardiomyopathy) and only Chagas disease (digestive form). The likelihood of receiving a heart transplant was calculated by dividing the annual number of heart transplants due to Chagas disease by the prevalence of Chagas disease with heart failure. The total number of heart transplants was obtained by the National Transplant System,^28^ with 30% attributed to Chagas disease.^29^

### Indirect costs

Indirect costs included only patients’ absenteeism defined as the number of workdays missed. Productivity losses related to job vacancies resulting from death were not included in the model due to the lack of data on vacancy duration in the context of chronic Chagas disease. Absenteeism parameters were sourced from the literature for each chronic Chagas disease form. The daily monetary value was calculated using the ratio of Gross Domestic Product (GDP) per capita to the number of working days in 2023 (Supplementary Table S2).

### Sensitivity analysis

Deterministic and probabilistic sensitivity analyses were performed for costs outputs. Initially, a deterministic analysis was used to replace key baseline model parameters, one at a time. Chagas disease prevalence estimates of 1.02% and 1.88% were drawn from Dias et al.^4^ and Laporta et al.,^6^ respectively. Two alternative initial patient distributions across chronic Chagas disease forms, according to different study settings (endemic countries and overall studies), were selected from a recent meta-analysis.^15^ The deterministic analysis also examined the impact of varying absenteeism costs, based on the average wage rate of the Brazilian workforce, and inpatient care costs, using reimbursement rates from the Ministry of Health for inpatient care delivered by the SUS. This latter simulation estimates a lower bound for inpatient care costs, as the Ministry of Health reimbursement rates do not fully cover expenses associated with hospitalizations funded by SUS.

Another deterministic sensitivity analysis was implemented to examine the effects of varying the baseline model parameters within specified intervals: transition probabilities (indeterminate to Chagas cardiomyopathy, indeterminate to digestive, and cardiac to death), using maximum and minimum limits from the literature, and DRG-Brasil inpatient costs (±20%). These results were presented in a tornado diagram. Finally, a probabilistic sensitivity analyses was conducted by simultaneously varying transition probabilities and costs parameters (inpatient care and absenteeism). Uniform distributions were used for transition probabilities, and triangular distributions were applied for costs. For the transition probability from indeterminate to cure, a beta distribution was assumed since enough information was available. Supplementary Table S8 details the parameters used in the sensitivity analysis.

### Role of the funding source

The funders had no role in study design, data collection and analysis, decision to publish, or preparation of the manuscript. All authors accept responsability to submit the paper for publication. This article represents the views of the authors and should not be interpreted as reflecting the views of their employers. The authors declare no further conflicts of interest.

## Results

### Descriptive analysis

Table 1 presents the average direct and indirect medical costs per patient calculated considering the proportion of patients receiving each procedure. Expected total medical direct costs varies significantly among the chronic Chagas disease forms. The lowest annual values are observed for the indeterminate form, $228, and the highest for the mixed form, $4944. Inpatient care represents the most important component mainly for the cardiac form, totaling $3984 per year, which includes surgical and clinical hospitalizations. The most expensive surgical procedures are implantable cardiac defibrillator implantation followed by heart transplant. Annual average costs for implantable cardiac defibrillator, including the value of the prosthesis, was estimated at $43,428 and performed for 19.4% of patients with Chagas cardiomyopathy that received surgical hospitalizations. Annually, 4.7% of Chagas cardiomyopathy patients in Brazil is hospitalized for surgical purposes. Annual average heart transplant costs per patient were estimated at $27,706, but the annual proportion of Chagas cardiomyopathy patients that receive heart transplant is extremely low, around 0.00012 (Supplementary Table S7).

**Table 1:** Average medical direct and indirect costs per chronic Chagas disease patient by cost components in Brazil (2024 purchasing power parity US dollars).

Periodicity	Direct medical cost				Indirect costs^b^
	Expected expenditure per patient^a^				
	Total	Outpatient care	Medicines	Inpatient care	
**Indeterminate form**					
First year^c^	7.22	–	7.22	–	**–**
Annual	227.94	227.94	**–**	**–**	455.40
**Cardiac form**					
First year^c^	651.15	651.15	**–**	**–**	**–**
Annual	4722.41	515.38	223.26	3983.77	1366.20
**Digestive form**					
First year^c^	583.72	583.72	**–**	**–**	**–**
Annual	1752.51	347.90	201.51	1203.10	1358.46
Twice in a lifetime^d^	193.17	193.17	**–**	**–**	**–**
**Mixed form**					
First year^c^	907.18	907.18	**–**	**–**	**–**
Annual	4944.13	535.60	424.77	3983.77	1366.20
Twice in a lifetime^d^	193.17	193.17	**–**	**–**	**–**

a Expected expenditure per patient was obtained multiplying the unit cost of each procedure by multiplying its frequency and the probability of utilization.

b Indirect costs consider only absenteeism.

c First year following the chronic Chagas disease diagnosis.

d It refers to procedures that, according to clinical practice guidelines and expert consensus are typically performed two times over the course of a patient’s life.

Expected outpatient costs range from $228 (indeterminate form) to $907 (mixed form) per patient per year. The highest values are observed in the first-year as they include diagnosis procedures. The first-year costs are estimated at $651 (cardiac form), $584 (digestive form), and $907 (mixed form) per patient, while subsequent annual costs are $515 and $348, for the cardiac and digestive forms, respectively. Beyond annual procedures, the digestive form requires specific exams and tests, such as simple abdominal X-rays and interventional digestive endoscopy, which were assumed to be performed twice over a patient’s lifetime ($193 per patient).

Medicines for the indeterminate form include only the etiological treatment in the first year ($7 per patient). Chagas cardiomyopathy includes drugs for treatment for heart failure and atrial fibrillation, with an expected annual cost of $223 per patient. In the digestive and mixed forms, costs are estimated at $202 and $425 per patient. Mixed form includes medicines for both Chagas cardiomyopathy and digestive forms.

Indirect costs were substantial reflecting the number of days that chronic Chagas disease prevents individuals from working activities. The highest annual values per patient were estimated for the cardiac and digestive forms, around $1300 per year, and lower for the indeterminate form, $439.

### Markov models

Table 2 presents the cost outputs related to chronic Chagas Disease in Brazil considering the prevalence of Chagas disease in 2010 for population aged 15 years and older. The lifetime economic burden is estimated at $45,033 per patient resulting in a total cost of $156.53 billion. The annual cost per patient was estimated at $3292 reaching a total annual cost of $11.44 billion, accounting for 0.23% of Brazil's GDP. Considering only direct medical costs, the total annual amount would represent 11% of the Ministry of Health's expenditures and 1.92% of total healthcare expenditures. Costs due to absenteeism were significant, corresponding to 28% of the average lifetime costs ($44 billion) and total annual costs ($3.17 billion). Considering only patients with cardiac and digestive forms, the annual average costs were $6126 and $3,723, with the total costs representing 0.14% and 0.07% of the GDP, respectively.

**Table 2:** Costs outputs estimates for chronic Chagas disease using Markov Model in Brazil.

Costs outputs	Total^a^ (billions)	Per patient^a^	GDP (%)^b^	MoH (%)^c^	Total Healthcare Expenditures (%)^c^	Economic Burden (%)
**Lifetime**^d^						
Economic burden^e^	156.53	45,034	–	–		–
Direct medical costs	112.53	32,376	–	–		71.89
Indirect costs^f^	43.99	12,658	–	–		28.11
**Annual**						
Economic burden^e^	11.44	3292	0.23	–		–
Direct medical costs	8.27	2380	0.17	10.81	1.92	72.28
Indirect costs^f^	3.17	913	–	–		27.72
Cardiac economic burden^e^	7.09	6126	0.14	–		–
Digestive economic burden^e^	3.51	3723	0.07	–		–

a Costs expressed in 2024 purchasing power parity US dollars (PPP-USD).

b Brazilian 2023 GDP converted to 2024 PPP-USD is $4905 billion.^30^

c Ministry of Health (MoH) expenditure $76.55 billion in 2023^31^ converted to 2024 PPP-USD and total healthcare expenditures in Brazil $430.65 billion in 2019 converted to 2024 PPP-USD.^32^

d Lifetime estimates consider 3% discount rate.

e Economic burden includes direct medical costs and indirect costs (absenteeism).

f Indirect costs consider only absenteeism.

The number of expected QALY was 20.6 per individual, rising to 31.5 years if chronic Chagas disease patients experienced general population mortality rates. These findings evidence 10.9 years of life lost due to chronic Chagas disease. The HRQoL loss is about 0.9 year per individual. Finally, the average patient with Chagas cardiomyopathy is expected to live around 11.8 years, representing a loss of 19.7 years compared to the scenario in which patients experience general mortality rates. For the digestive form, these figures were 23.7 and 7.8 respectively (Table 3).

**Table 3:** Markov Model health outputs estimates for adult population (15+) with chronic Chagas disease (CCD).

**Expected Quality-Adjusted Life Years per patient with CCD**	
All Forms (A)	20.6
Cardiac form	11.8
Digestive form	23.7
**Expected life years per patient with CCD (without disability weights)**	
All Forms (B)	21.5
**Expected quality-adjusted life years per patient (assuming general population mortality rates)**	
All Forms (C)	31.5
**Years of life lost and HRQoL loss due to CCD**	
Years of life lost due to CCD (C-A)	10.9
HRQoL loss due to CCD (B-A)	0.9

HRQoL, Health Related Quality of Life.

### Sensitivity analysis

The lifetime economic burden proved most sensitive to variations in Chagas disease prevalence and inpatient care costs. As expected, reducing the prevalence to 1.02% would yield a 57.5% reduction in economic burden. When using Ministry of Health inpatient care reimbursement rates, the economic burden ranges from $156.53 billion (baseline) to $90.61 billion, reflecting a 42% decrease. The level of endemicity also influenced the lifetime economic burden. Assuming the initial distribution of patients across chronic Chagas disease forms as observed in endemic countries, which represent a greater weight on cardiac and mixed forms, the economic burden would increase to $167 billion, representing a 6.9% rise (Table 4).

**Table 4:** Deterministic sensitivity analysis for costs outputs (Total in billions) for chronic Chagas disease (CCD) in Brazil (2024 purchasing power parity US dollars).

Sensitivity analysis	Lifetime^a^			Annual		
	Economic Burden^b^	Direct Medical Costs	Indirect costs^c^	Economic Burden^b^	Direct Medical Costs	Indirect costs^c^
Baseline model	**156.53**	**112.53**	**44.00**	**11.44**	**8.27**	**3.17**
**Prevalence**						
1.88%	122.61	88.15	34.46	8.96	6.48	2.49
1.02%	66.52	47.83	18.70	4.86	3.52	1.35
**Initial patient distribution across CCD Forms**						
Overall studies	161.30	118.71	42.59	12.48	9.20	3.29
Endemic countries	166.99	123.59	43.40	13.16	9.75	3.41
**Costs**						
Inpatient^d^	90.61	46.61	44.00	6.58	3.40	3.17
Absenteeism^e^	146.07	112.53	33.54	10.69	8.27	2.42

a Lifetime estimates consider 3% discount rate.

b Economic burden includes direct medical costs and indirect costs (absenteeism).

c Indirect costs consider only absenteeism.

d Using inpatient cost parameters from Hospital Information System (SIH-DATASUS) 2022.

e Using the average monthly wage reported by 2023 Continuous National Household Sample Survey (PNADC) to monetize absenteeism.

**Fig. 3:**
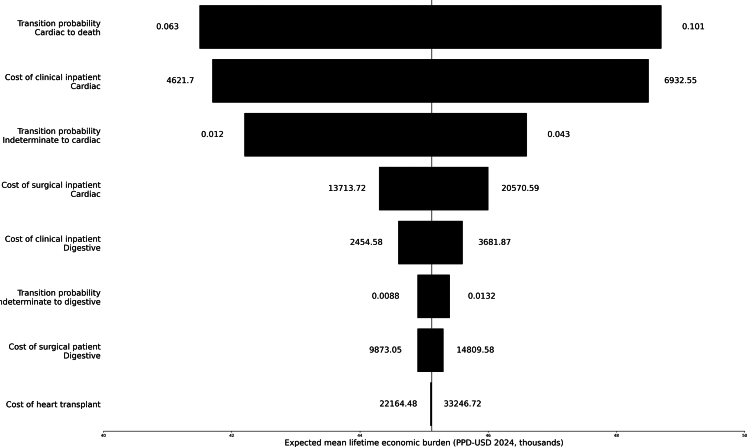
**Effect of varying transition probabilities and inpatient costs on lifetime economic burden of chronic Chagas disease per patient in Brazil**.

Fig. 3 shows the tornado diagram regarding the effect of varying each transition probability and inpatient care costs. The lifetime economic burden per patient was most sensitive to changes in parameters associated with the cardiac form.

The probabilistic sensitivity analysis shows that the total lifetime economic burden ranges from $119.1 billion to $192.9 billion, representing a 24% decrease and a 23% increase relative to the baseline outcome. Lifetime direct medical costs range from $82.9 billion to $141.8 billion, reflecting a 26% decrease and 26% increase. The interval with the 95% of iterations spans from $132.8 to $175.2 for the lifetime economic burden and from $93.4 to $127.5 for lifetime direct medical costs.

## Discussion

This study provides a comprehensive estimation of the economic burden of chronic Chagas disease in Brazil from a societal perspective, a robust approach that differs from previously published analyses. A cost-of-illness framework was selected for its appropriateness in estimating disease-specific economic burden, as it enables the disaggregation of costs by clinical form and cost component. This level of detail is particularly relevant for chronic Chagas disease, a condition characterized by heterogeneous clinical manifestations and a prolonged disease course that are effectively captured through a Markov microsimulation model. While alternative approaches based on macroeconomic models are useful for assessing broader economic impacts, such as GDP losses through general equilibrium effects, they are less suited to capturing the structural and distributional aspects of disease burden. These models are commonly applied to evaluate the economic implications of health shocks mainly related to outbreaks of diseases but do not provide the granularity needed for condition-specific burden analyses.^33^^,^^34^

The main findings indicate that the annual economic burden of chronic Chagas disease is approximately $11.44 billion, constituting a significant proportion of Brazil's GDP (0.23%). When focusing solely on direct medical costs, these expenditures account for 11% of the Ministry of Health budget and 1.92% of total health expenditures. Annual medical costs per patient is estimated at $2380 which exceeds the total health expenditure per capita in Brazil ($2049.30^32^). The lifetime economic burden per patient, which reflects the expected costs incurred throughout an individual's life cycle, is notably substantial at $45,034, nearly double the Brazilian GDP per capita. These estimations are based on the prevalence of Chagas disease among the Brazilian population aged 15 years and older as of 2010, thus representing current costs associated with chronic Chagas disease, given that most of these patients remain alive. Furthermore, although the incidence of Chagas disease has been declining in Brazil – primarily due to socioeconomic advancements – this disease has not been eliminated, and new infections have been documented since 2010.

Chagas cardiomyopathy incurs the highest economic burden, amounting to $7.09 billion, which corresponds to an annual average burden of $6126 per patient. In comparison, the economic burden of the digestive form is estimated at $3.51 billion, with an average cost of $3723 per patient. Previous estimates of the economic burden of cardiac diseases also indicated a significant impact on the Brazilian healthcare budget. Stevens et al.^35^ found that the direct medical costs associated with cardiac diseases in 2015 amounted to 5.5% of total Brazilian health expenditures, assuming that 32% of the adult population had some cardiac condition. These figures were notably higher when considering only severe cases (with a prevalence of 5.2% of the population aged over 35 years), reaching 8% of the national health expenditures.^36^

Indirect costs play a crucial role in assessing the overall economic burden of chronic Chagas disease. In this analysis, absenteeism due to lost workdays constituted a significant portion of the economic burden, accounting for approximately one third. This result is conservative, as it does not account for other factors such as productivity losses associated with presenteeism - when employees are present at work but are not fully productive due to health issues. Similar results regarding the share of indirect costs in total economic burden of Chagas disease were also found by Oliveira and Buitrago^8^ for Colombia and Lee et al.^9^ for the global economic burden. The total years of life lost due to Chagas disease are substantial, averaging 10.9 years per individual and reaching 19.7 years for patients with the cardiac form of the disease.

The Brazilian institutional context offers valuable insights for addressing universal healthcare coverage in middle- and low-income countries. Chronic conditions requiring long-term care, such as chronic Chagas disease, entail substantial costs that can place a significant strain on family budgets. In this regard, an organized universal healthcare system is a crucial buffer against the potentially catastrophic financial burdens of chronic Chagas disease treatment. In Brazil, universal healthcare coverage is especially vital given the pronounced socioeconomic disparities across individuals and regions. Without the availability of SUS as a universal public coverage, many chronic Chagas disease patients would likely face catastrophic expenditures, exacerbating their vulnerability to poverty.

Studies that evaluate the economic burden of Chagas disease from the societal perspective are still scarce worldwide.^7^, ^8^, ^9^, ^10^ The estimation presented in this study accounts for both direct medical costs and indirect costs associated with absenteeism. The direct medical costs include all procedures that patients with chronic Chagas disease are expected to undergo throughout their lifetime following a diagnosis of Chagas disease, considering the odds for developing each of Chagas disease clinical forms.

The most comprehensive study was conducted by Lee et al.^9^ in an analysis aiming to estimate global economic burden considering 33 countries with incidence of Chagas disease. Figures of the present study align with estimates from Lee et al.^9^ for Latin America, $517, and for the US, Canada, and Australia, $2916 (values updated to 2024 purchasing power parity). This study advances upon Lee et al.'s estimation for Brazil in three key ways. First, the definition of outpatient care procedures and medications was based on official protocols validated by clinical experts, while hospitalizations were sourced from the DRG-Brasil database using ICD-10 codes related to Chagas disease. Second, all cost values were derived from real-world data, ensuring an accurate representation of healthcare expenditures within the Brazilian Healthcare System. Finally, the probability of receiving care was determined using a national household health survey that enabled to investigate individuals with Chagas disease.

Studies estimating the economic costs of Chagas disease in Brazil are quite limited and focused on specific institutions.^37^^,^^38^ Abuhab et al.^38^ assessed data from a high-complexity cardiology university hospital in São Paulo and found that the expected total cost per event for patients with Chagas cardiomyopathy during acute decompensated heart failure admissions was approximately $5174 (updated to 2024 purchasing power parity). These estimates are higher than the costs identified in this study for the inpatient care of Chagas cardiomyopathy patients, which encompasses both surgical and clinical hospitalizations. Hasslocher-Moreno et al.^37^ employed a micro-costing approach to estimate all expenses associated with the Laboratory of Clinical Research in Chagas Disease at the Fiocruz Foundation in Brazil. As their results were not reported on a per-patient or per-episode basis, direct comparisons are not feasible.

This study presents some limitations. First, direct non-medical costs, such as transportation, accommodation, and food expenses, were not included in the analysis. Estimating these expenditures typically requires field research with patients or, at a minimum, clinical records that contain residential address information or geographical heatmaps of utilization. Given that this analysis was conducted at the national level, the inclusion of this component was not feasible due to budgetary constraints. An estimate for Colombia – the only study available for Latin American countries – derived from a 2017 field survey of patients with Chagas disease, indicated that non-medical direct costs represented 20.4% of the total direct costs.^8^ Assuming a similar share of non-medical direct costs in Brazil, the total annual direct cost would amount to $9.19 billion, representing 0.19% of the country’s GDP, compared to 0.17% when only medical direct costs were considered.

Secondly, the estimation method assessed the total value of healthcare services without identifying whether these costs were borne by families or covered by the healthcare system (SUS or private health insurance). In Brazil, SUS provides universal and comprehensive healthcare to all residents. However, access to secondary care services - such as imaging exams, specialist consultations, and medications - is often limited. As a result, families may seek for care in the private sector, either through health insurance or by paying out-of-pocket expenses. Although around 30% of the Brazilian population is covered by private health insurance, families frequently incur additional costs for copayments and medication. Furthermore, since Chagas disease patients generally belong to lower socioeconomic groups, the private insurance coverage is probably less common among them. These disparities, compounded by Brazil's persistent issues with inequality and poverty, underscore an important avenue for future research on Chagas disease.

Finally, certain parameters, specifically the transition probabilities and the days of absenteeism, were not available within the Brazilian context. These parameters were sourced from the literature, including two meta-analyses. Notably, the majority of the selected studies in these meta-analyses were conducted in Brazil^39^^,^^40^ and these parameters were validated by clinical experts. A further consideration is that some healthcare utilization parameters are not specific to patients with Chagas disease, particularly those with the digestive and indeterminate forms, which may potentially jeopardize the external validity of our findings. To address this, for the indeterminate form, as patients are generally asymptomatic and require limited medical intervention, healthcare utilization parameters were approximated using the probability observed for the general population, thereby reflecting the access conditions within the Brazilian Health System. In the absence of data on digestive conditions within the household survey, healthcare utilization for the digestive form relied solely on reports from individuals with Chagas disease. Conversely, for patients with the cardiac form, healthcare utilization parameters were directly retrieved from individuals who reported both Chagas disease and cardiac conditions.

To the best of our knowledge, this is the first study to evaluate the economic burden of chronic Chagas disease at the national level in Brazil, considering a societal perspective, with a comprehensive assessment of costs. This research further estimates cost parameters for different forms of Chagas disease, allowing for the disaggregation of costs into categories such as inpatient and outpatient care, as well as medications, based on real-world data. The estimations presented in this paper represent a significant advancement in supporting cost-effectiveness analyses of new technologies related to the treatment, prevention, and screening of Chagas disease. Furthermore, it contributes to the allocation and prioritization of health resources. Given that Chagas disease is a preventable condition, the economic burden data allow for estimating potential savings from averted cases, thereby enabling more efficient resource allocation and customized planning of specific health policies.

## Contributors

Conceptualization: MVA, KVMSN, AS, ASMS, ALPR; Data curation: MVA, KVMSN, AS, NAJ, ASMS, PEFB, HB, YCS, BRN, MC, FRMM, IEM, ALPR; Formal analysis: MVA, KVMSN, AS, NAJ, ASMS, PEFB, HB, YCS, BRN, MC, FRMM, IEM, PP, YG, CD, ALPR; Funding acquisition: MVA, ALPR; Investigation: MVA, KVMSN, AS, NAJ, ASMS, PEFB, HB, YCS; Methodology: MVA, KVMSN, AS, NAJ, ASMS, PEFB, HB, YCS; Project administration: MVA, KVMSN, AS, ALPR; Resources: PP, YG, CD, ALPR; Supervision: MVA, KVMSN; Validation: MVA, KVMSN, AS, NAJ, ASMS, PEFB, HB, YCS, BRN, MC, FRMM, IEM, PP, YG, CD, ALPR; Visualization: AS, NAJ, ASMS, PEFB, HB; Writing – original draft: MVA, KVMSN, AS, NAJ; Writing – review & editing: MVA, KVMSN, AS, NAJ, ASMS, PEFB, HB, YCS, BRN, MC, FRMM, IEM, PP, YG, CD, ALPR. All authors read and approved the final version for submission.

## Data sharing statement

Upon reasonable request to the corresponding author, study data and analytical methods may be made available to other researchers for the purposes of reproducing the results or replicating the procedures of this study.

## Declaration of interests

P.P. is World Heart Federation employee. Y.G. and C.D. are Novartis Pharma AG employees. This article represents the views of the authors and should not be interpreted as reflecting the views of their employers. The authors declare no further conflicts of interest.

## Appendix A Supplementary data

Supplementary Tables S1–S8

Abstract-portuguese
