# Supplementary material for: Economic burden of Chagas disease in Brazil: a nationwide cost-of-illness study
Source: Lancet Reg Health Am. 2025 Aug 8;50:101202. doi: 10.1016/j.lana.2025.101202 (PMC12356041; doi:10.1016/j.lana.2025.101202)
Supplement: Supplementary Tables S1–S8 [file mmc1.docx]

**Economic burden of Chagas disease in Brazil: a nationwide cost-of-illness study**

**Supplementary Material**

Summary

Supplementary Table 1: Consolidated Health Economic Evaluation Reporting Standards 2022 (CHEERS 2022)2

Supplementary Table 2: Indirect costs due to absenteeism and Global Burden Disease (GBD) weight sequalae4

Supplementary Table 3: Transition probabilities among health states5

Supplementary Table 4: General mortality rate for population over 14 years old for each cycle6

Supplementary Table 5: Outpatient Care Costs for each Chagas disease clinical form8

Supplementary Table 6: Medicines posology and costs for each Chagas disease Clinical form9

Supplementary Table 7: Cost parameters for inpatient care for each Chagas disease form10

Supplementary Table 8: Sensitivity analysis parameters11

References12

Supplementary Table 1: Consolidated Health Economic Evaluation Reporting Standards 2022 (CHEERS 2022^1^) Checklist.

| **Topic** | **No.** | **Item** | **Location where item is reported** |
| --- | --- | --- | --- |
| **Title** |  |  |  |
|  | 1 | Identify the study as an economic evaluation and specify the interventions being compared. | Title, Page 1 |
| **Abstract** |  |  |  |
|  | 2 | Provide a structured summary that highlights context, key methods, results, and alternative analyses. | Abstract, Page 1 |
| **Introduction** |  |  |  |
| **Background and objectives** | 3 | Give the context for the study, the study question, and its practical relevance for decision making in policy or practice. | Introduction, 4^th^ and 5^th^ Paragraphs |
| **Methods** |  |  |  |
| **Health economic analysis plan** | 4 | Indicate whether a health economic analysis plan was developed and where available. | Not Reported |
| **Study population** | 5 | Describe characteristics of the study population (such as age range, demographics, socioeconomic, or clinical characteristics). | Methods, 2^nd^ subsection 2^nd^ and 4^th^ Paragraphs |
| **Setting and location** | 6 | Provide relevant contextual information that may influence findings. | Methods, 1^st^ subsection; Methods, 4^t^ subsection |
| **Comparators** | 7 | Describe the interventions or strategies being compared and why chosen. | Not Applicable |
| **Perspective** | 8 | State the perspective(s) adopted by the study and why chosen. | Methods, 1^st^ subsection |
| **Time horizon** | 9 | State the time horizon for the study and why appropriate. | Methods, 2^nd t^ subsection 2^nd^ Paragraph |
| **Discount rate** | 10 | Report the discount rate(s) and reason chosen. | Methods, 2^nd^ subsection 4^th^ Paragraph |
| **Selection of outcomes** | 11 | Describe what outcomes were used as the measure(s) of benefit(s) and harm(s). | Not Applicable |
| **Measurement of outcomes** | 12 | Describe how outcomes used to capture benefit(s) and harm(s) were measured. | Not Applicable |
| **Valuation of outcomes** | 13 | Describe the population and methods used to measure and value outcomes. | Not Applicable |
| **Measurement and valuation of resources and costs** | 14 | Describe how costs were valued. | Methods, 4^th^ and 5^th^ subsections |
| **Currency, price date, and conversion** | 15 | Report the dates of the estimated resource quantities and unit costs, plus the currency and year of conversion. | Methods, 1^st^ subsection; Supplementary Material, Tables S2 and S5-S7 |
| **Rationale and description of model** | 16 | If modelling is used, describe in detail and why used. Report if the model is publicly available and where it can be accessed. | Methods, 2^nd^ subsection |
| **Analytics and assumptions** | 17 | Describe any methods for analysing or statistically transforming data, any extrapolation methods, and approaches for validating any model used. | Methods and Supplementary Material |
| **Characterising heterogeneity** | 18 | Describe any methods used for estimating how the results of the study vary for subgroups. | Not Applicable |
| **Characterising distributional effects** | 19 | Describe how impacts are distributed across different individuals or adjustments made to reflect priority populations. | Not Applicable |
| **Characterising uncertainty** | 20 | Describe methods to characterise any sources of uncertainty in the analysis. | Methods, 6^th^ subsection |
| **Approach to engagement with patients and others affected by the study** | 21 | Describe any approaches to engage patients or service recipients, the general public, communities, or stakeholders (such as clinicians or payers) in the design of the study. | Not Applicable |
| **Results** |  |  |  |
| **Study parameters** | 22 | Report all analytic inputs (such as values, ranges, references) including uncertainty or distributional assumptions. | Results, 1^st^ subsection and Supplementary Material |
| **Summary of main results** | 23 | Report the mean values for the main categories of costs and outcomes of interest and summarise them in the most appropriate overall measure. | Results, 2^nd^ subsection |
| **Effect of uncertainty** | 24 | Describe how uncertainty about analytic judgments, inputs, or projections affect findings. Report the effect of choice of discount rate and time horizon, if applicable. | Results, 3^rd^ subsection |
| **Effect of engagement with patients and others affected by the study** | 25 | Report on any difference patient/service recipient, general public, community, or stakeholder involvement made to the approach or findings of the study | Not Applicable |
| **Discussion** |  |  |  |
| **Study findings, limitations, generalisability, and current knowledge** | 26 | Report key findings, limitations, ethical or equity considerations not captured, and how these could affect patients, policy, or practice. | Discussion |
| **Other relevant information** |  |  |  |
| **Source of funding** | 27 | Describe how the study was funded and any role of the funder in the identification, design, conduct, and reporting of the analysis | Acknowledgments |
| **Conflicts of interest** | 28 | Report authors conflicts of interest according to journal or International Committee of Medical Journal Editors requirements. | Declaration of interests |

Supplementary Table 2: Indirect costs due to absenteeism and Global Burden Disease (GBD) weight sequalae**.**

| **Clinical forms** | **Parameter** | **Value ^(3)^** | **Percentage of patients** | **Sources** |
| --- | --- | --- | --- | --- |
| **Indeterminate** | Absenteeism ^(1)^ | 5 days | 1·00 | Lee et al (2013)^2^ |
| **Cardiac** | Weight sequalae ^(2)^ – Heart Failure | 0·041 (mild) | 0·246 | SaMi-Trop^3^ and Panel of experts |
|  | Weight sequalae ^(2)^ – Heart Failure | 0·072 (moderate) | 0·246 | SaMi-Trop^3^ and Panel of experts |
|  | Weight sequalae ^(2)^ – Heart Failure | 0·179 (severe) | 0·246 | SaMi-Trop^3^ and Panel of experts |
|  | Weight sequalae ^(2)^ – Atrial Fibrillation | 0·224 | 0·184 | SaMi-Trop^3^ and Panel of experts |
|  | Absenteeism ^(1)^ | 15 days | 1·00 | Lee et al (2013)^2^ and Panel of experts |
| **Digestive** | Weight sequalae ^(2)^ – Abdominopelvic problem | 0·011 (mild) | 1·00 | Panel of experts |
|  | Weight sequalae ^(2)^ – Abdominopelvic problem | 0·114 (moderate) | 1·00 | Panel of experts |
|  | Absenteeism ^(1)^ | 11 days | 1·00 | Lee et al (2013)^2^ and Panel of experts |
|  | Weight sequalae ^(2)^ – Abdominopelvic problem | 135 days (surgery) | 0·029 (surgery) | Lee et al (2013)^2^, IBGE (2020)^4^ |
| **Mixed** | Weight sequalae ^(2,4)^ – Heart Failure | 0·041 (mild) | 0·246 | SaMi-Trop^3^ and Panel of experts |
|  | Weight sequalae ^(2,4)^ – Heart Failure | 0·072 (moderate) | 0·246 | SaMi-Trop^3^ and Panel of experts |
|  | Weight sequalae ^(2,4)^ – Heart Failure | 0·179 (severe) | 0·246 | SaMi-Trop^3^ and Panel of experts |
|  | Weight sequalae ^(2,4)^ – Atrial Fibrillation | 0·224 | 0·184 | SaMi-Trop^3^ and Panel of experts |
|  | Absenteeism ^(1)^ | 15 days | 1·00 | Lee et al (2013)^2^ and Panel of experts |
| Note: (1) The cost of absenteeism was calculated by dividing the 2023 per capita Gross Domestic Product of R$50,193·72 (Purchasing Parity Power US dollar (PPP-USD) $22,587·17) by the 248 working days in 2023 (PPP-USD 91·08 in 2024). (2) Weight sequalae from Global Burden Disease (2019).^5^ (3) According to a panel of experts, for weight sequalae, patients were equally distributed among the levels of severity (mild/moderate/severe) when applicable. (4) The parameters for the mixed form are assumed to be the same as cardiac form. | | | | |

Supplementary Table 3: Transition probabilities among health states.

| **From:** | **To:** | **Parameter** | **Source** |
| --- | --- | --- | --- |
| **Indeterminate** | Cardiac | 0·03 | Chadalawada et al. (2020)^6^ |
|  | Digestive | 0·011 | Castro et al. (1994)^7^ |
|  | Death ^(1)^ | General mortality rate (see Table S4) | IBGE (2011)^8^ |
|  | Cure | 0·08 | Lee et al. (2013)^2^, Andrade et al. (1996)^9^, Ferreira (1990)^10^ |
| **Cardiac** | Mixed | 0·011 | Castro et al. (1994)^7^ |
|  | Death | 0·079 | Chadalawada et al. (2021)^11^ |
| **Digestive** | Mixed | 0·03 | Chadalawada et al. (2020)^6^ |
|  | Death | 0·0225*0·029 + General mortality rate ^(2)^ | Lee et al (2013)^2^, Wilson et al. (2005)^12^ |
| **Mixed** | Death | 0·079 ^(3)^ | Chadalawada et al. (2021)^11^ |
| Note: (1) General mortality rate for population over 14 years old for each cycle. (2) Parameter of 0·0225 represents the mortality probability for patients undergoing megacolon surgery. Probability of 0·029 reflects the likelihood of surgical hospitalization among patients with Chagas disease, as reported by the National Health Survey (IBGE, 2020).^4^ (3) The probability of mortality from the mixed form was determined by selecting the higher value between the cardiac and digestive forms. | | | |

Supplementary **Table 4: General mortality rate for population over 14 years old for each cycle**.

| **Age** | **Parameter** |
| --- | --- |
| 15 years or older | 0·01618 |
| 16 years or older | 0·01644 |
| 17 years or older | 0·01670 |
| 18 years or older | 0·01697 |
| 19 years or older | 0·01724 |
| 20 years or older | 0·01751 |
| 21 years or older | 0·01780 |
| 22 years or older | 0·01809 |
| 23 years or older | 0·01839 |
| 24 years or older | 0·01870 |
| 25 years or older | 0·01903 |
| 26 years or older | 0·01936 |
| 27 years or older | 0·01971 |
| 28 years or older | 0·02007 |
| 29 years or older | 0·02045 |
| 30 years or older | 0·02083 |
| 31 years or older | 0·02124 |
| 32 years or older | 0·02166 |
| 33 years or older | 0·02210 |
| 34 years or older | 0·02255 |
| 35 years or older | 0·02303 |
| 36 years or older | 0·02352 |
| 37 years or older | 0·02404 |
| 38 years or older | 0·02457 |
| 39 years or older | 0·02513 |
| 40 years or older | 0·02572 |
| 41 years or older | 0·02633 |
| 42 years or older | 0·02697 |
| 43 years or older | 0·02763 |
| 44 years or older | 0·02833 |
| 45 years or older | 0·02906 |
| 46 years or older | 0·02982 |
| 47 years or older | 0·03061 |
| 48 years or older | 0·03145 |
| 49 years or older | 0·03232 |
| 50 years or older | 0·03324 |
| 51 years or older | 0·03421 |
| 52 years or older | 0·03523 |
| 53 years or older | 0·03631 |
| 54 years or older | 0·03744 |
| 55 years or older | 0·03863 |
| 56 years or older | 0·03989 |
| 57 years or older | 0·04122 |
| 58 years or older | 0·04262 |
| 59 years or older | 0·04410 |
| 60 years or older | 0·04567 |
| 61 years or older | 0·04734 |
| 62 years or older | 0·04911 |
| 63 years or older | 0·05099 |
| 64 years or older | 0·05299 |
| 65 years or older | 0·05512 |
| 66 years or older | 0·05738 |
| 67 years or older | 0·05978 |
| 68 years or older | 0·06234 |
| 69 years or older | 0·06508 |
| 70 years or older | 0·06803 |
| 71 years or older | 0·07120 |
| 72 years or older | 0·07462 |
| 73 years or older | 0·07831 |
| 74 years or older | 0·08228 |
| 75 years or older | 0·08654 |
| 76 years or older | 0·09111 |
| 77 years or older | 0·09600 |
| 78 years or older | 0·10126 |
| 79 years or older | 0·10694 |
| 80 years or older | 0·11309 |
| 81 years or older | 0·11979 |
| 82 years or older | 0·12704 |
| 83 years or older | 0·13482 |
| 84 years or older | 0·14307 |
| 85 years or older | 0·15164 |
| 86 years or older | 0·16041 |
| 87 years or older | 0·16932 |
| 88 years or older | 0·17841 |
| 89 years or older | 0·18787 |
| 90 years or older | 0·19798 |
| Source: Brazilian Institute of Geography and Statistics (IBGE, 2011).^8^ | |

Supplementary Table 5: Outpatient Care Costs for each Chagas disease clinical form**.**

| **Clinical forms** | **Procedures** | **Frequency ^(1)^** | **Unit cost ^(2)^** | **Individuals using health services** | |
| --- | --- | --- | --- | --- | --- |
|  |  |  | **(PPP-USD 2024)** | **Proportion** | **Source** |
| **Indeterminate** | Electrocardiogram | 1 (per year) | 20·85 | 1 | Protocols |
|  | Doctor visits | 3·81 (per year) | 67·94 | 0·8 | IBGE (2020)^4^ |
| **Cardiac** | Holter | 1 (per year) | 94·96 | 0·1 | Panel of experts |
|  | Electrocardiogram | 1 (per year) | 20·85 | 1 | Protocols |
|  | Echocardiogram | 1 (at diagnosis) | 196·44 | 1 | Protocols |
|  | Echocardiogram | 1 (every 3 years) | 65·48 | 1 | Protocols |
|  | Exercise stress test | 1 (per year) | 50·43 | 0·05 | Panel of experts |
|  | X-ray | 1 (at diagnosis) | 48·12 | 0·1 | Panel of experts |
|  | Doctor visits | 6·2 (per year) | 67·94 | 0·99 | IBGE (2020)^4^ |
| **Digestive** | Contrast X-ray of the esophagus | 1 (at diagnosis) | 48·12 | 1 | Panel of experts |
|  | Contrast X-ray of the esophagus | 1 (every 3 years) | 16·04 | 0·5 | Panel of experts |
|  | Simple abdominal X-ray | Twice in a lifetime | 48·12 | 0·17 | Panel of experts |
|  | Contrast X-ray of the colon | 1 (at diagnosis) | 48·12 | 1 | Panel of experts |
|  | Contrast X-ray of the colon | 1 (every 3 years) | 16·04 | 0·17 | Panel of experts |
|  | Computed tomography (CT) | 2 (at diagnosis) | 162·28 | 0·17 | Panel of experts |
|  | Magnetic resonance imaging (MRI) | 1 (at diagnosis) | 277·24 | 0·03 | Panel of experts |
|  | Colonoscopy | 1 (at diagnosis) | 252·8 | 0·33 | Panel of experts |
|  | Esophageal manometry | 1 (at diagnosis) | 61·1 | 0·17 | Panel of experts |
|  | Anorectal manometry | 1 (at diagnosis) | 82·72 | 0·03 | Panel of experts |
|  | Doctor visits | 5·3 (per year) | 67·94 | 0·91 | IBGE (2020)^4^ |
|  | Fecal impaction (manual removal) | 1 (every 2 years) | 111·36 | 0·17 | Panel of experts |
|  | Interventional digestive endoscopy | Twice in a lifetime | 267·9 | 0·33 | Panel of experts |
| **Mixed** | **Cardiac procedures + Digestive procedures** | **-** | **-** | **-** |  |
| Note: (1) Frequencies defined by protocols (PAHO, Brazilian Consensus on Chagas Disease, WHF IASC Roadmap on Chagas Disease, Therapeutic Guidelines (PCDT) for Chagas disease in Brazil)^13–16^ and a panel of experts. (2) Cost extracted from: Observatory of Hospital Policy and Management (FIOCRUZ) <http://tabnet.fiocruz.br/dhx.exe?ssuple/fat_internacao_procedim.DEF>^17^ referring to procedures done in hospitalizations in the private healthcare sector in December 2022, except price of Esophageal manometry, which was directly collected from Exchange of Information on Supplementary Health (TISS) in 2022. | | | | | |

Supplementary Table 6: Medicines posology and costs for each Chagas disease Clinical form.

| **Clinical forms** | **Medicines** | **Posology ^(1)^** | **Dose (cp)** | **Probability of taking the medicine ^(2)^** | **Unit cost (median) ^(3)^**  **(PPP-USD 2024)** | **Annual cost**  **(PPP-USD 2024)** | **Observation** |
| --- | --- | --- | --- | --- | --- | --- | --- |
| **Indeterminate** | Benznidazole | 5mg/kg/day (60 days) | 3·5 | **1·00** | 0·03 | **7·2** |  |
| **Cardiac** | **ACEI/ARB** |  |  | **0·185** |  | **11·8** | Probability considering that 3/4 of patients with heart failure (prevalence of 24·6%) use ACEI/ARB |
|  | Captopril | 75mg/day (lifetime) | 1·5 |  | 0·02 | 11·8 |  |
|  | Enalapril | 20mg/day (lifetime) | 1 |  | 0·04 | 15·7 |  |
|  | Losartan | 50mg/day (lifetime) | 1 |  | 0·02 | 7·8 |  |
|  | **Beta-blocker** |  |  | **0·246** |  | **42·4** | Probability considering the prevalence of heart failure among those with Chagas cardiomyopathy |
|  | Atenolol | 50mg/day (lifetime) | 1 |  | 0·02 | 7·8 |  |
|  | Metoprolol | 100mg/day (lifetime) | 1 |  | 0·09 | 34·5 |  |
|  | Carvedilol | 37·5mg/day (lifetime) | 1 |  | 0·09 | 33·0 |  |
|  | Bisoprolol | 10mg/day (lifetime) | 1 |  | 0·26 | 94·2 |  |
|  | **Spironolactone** | 25mg/day (lifetime) | 1 | **0·164** | 0·08 | **29·8** | Probability considering that 2/3 of patients with heart failure (prevalence of 24·6%) use Spironolactone |
|  | **Loop diuretic**  (Furosemide) | 40mg/day (lifetime) | 1 | **0·246** | 0·02 | **7·8** | Probability considering the prevalence of heart failure among those with Chagas cardiomyopathy |
|  | **Sacubitril + Valsartan** | 49mg+51mg/day (lifetime) | 1 | **0·062** | 1·72 | **626·2** | Probability considering that 1/4 of patients with heart failure (prevalence of 24·6%) replace ACEI/ARB with Sacubitril + Valsartan |
|  | **Dapagliflozin** | 10mg/day (lifetime) | 1 | **0·246** | 1·63 | **593·3** | Probability considering the prevalence of heart failure among those with Chagas cardiomyopathy |
|  | **Digitalis**  (Digoxin) | 0·125mg/day (lifetime) | 1 | **0·049** | 0·08 | **28·3** | Probability considering that 1/5 of patients with heart failure (prevalence of 24·6%) are refractory to quadruple therapy |
|  | **Antiarrhythmics**  **(**Amiodarone) | 200mg/day (lifetime) | 1 | **0·050** | 0·17 | **61·2** | Probability estimated by panel of experts |
|  | **Warfarin** | 5mg/day (lifetime) | 1 | **0·123** | 0·06 | **20·4** | Probability considering that 2/3 of patients with atrial fibrillation (prevalence of 18·4%) use Warfarin |
|  | **Direct Oral Anticoagulants (DOAC)** |  |  | **0·061** |  | **198·5** | Probability considering that 1/3 of patients with atrial fibrillation (prevalence of 18·4%) use DOAC |
|  | Rivaroxaban | 15mg/day (lifetime) | 1·5 |  | 0·20 | 108·3 |  |
|  | Apixaban | 10mg/day (lifetime) | 2 |  | 0·40 | 288·8 |  |
| **Digestive** | Laxatives (Bisacody) | 5mg/day (lifetime) | 1 | **0·500** | 0·43 | **157·0** |  |
|  | Suppository | Glycerol 95%/day (lifetime) | 1 | **0·250** | 0·61 | **224·4** |  |
|  | Enema (Sodium phosphate) | 1 every 7 days | 1 | **0·250** | 4·24 | **221·3** |  |
|  | Nifedipine | 30mg/ day (lifetime) | 1 | **0·0975** | 0·13 | **47·1** | Price: 1 tablet of 20mg + 1 tablet of 10mg |
|  | Isosorbide | 15mg/day (lifetime) |  | **0·048** | 0·40 | **146·0** | Price: 3 tablets of 5mg each (sublingual) |
| **Mixed** | **Cardiac medicines + Digestive medicines** |  |  |  |  |  |  |
| Note: (1) Doses considering the average weight of an adult of 70 kg. Frequencies defined by protocols (PAHO, Brazilian Consensus on Chagas Disease, WHF IASC Roadmap on Chagas Disease, Therapeutic Guidelines (PCDT) for Chagas disease in Brazil)^13–16^ and a panel of experts. (2) Probabilities from SaMi-Trop^3^ and a panel of experts. (3) Drug costs from the Health Price Panel - Brazilian Ministry of Health, except for the price of Benznidazole, which refers to the factory price set by the Drug Market Regulation Chamber (CMED)^18^ and price of Bisacody, which refers to the average price sold by drugstores. All drug cost data refer to the base year 2024. | | | | | | | |

Supplementary Table 7: Cost parameters for inpatient care for each Chagas disease form.

|  | **Average Cost** | | **Proportion of individuals that received inpatient care** | | |
| --- | --- | --- | --- | --- | --- |
|  | **N (patients) ^(4)^** | **Mean Cost**  **(PPP-USD 2024)** | **Frequency** | **Probability** | **Source** |
| **Surgical inpatient care for Cardiac Chagas disease ^(1)^** | **531** | **17,142·16** | **1 (per year)** | **0·047** | IBGE (2020)^4^ |
| Permanent Cardiac Peacemaker implantation | 366 | 11,846·81 |  |  |  |
| Cardiac defibrillator implantation without cardiac catheterization | 103 | 43,428·40 ^(5)^ |  |  |  |
| Review of Cardiac Pacemaker without replacement | 62 | 4,732·67 |  |  |  |
| **Clinical inpatient care for Cardiac Chagas disease ^(1)^** | **1304** | **5,777·12** | **2·13 (per year)** | **0·258** | IBGE (2020)^4^ |
| Heart failure and shock | 671 | 6,213·37 |  |  |  |
| Cardiac Arrhythmia and Conduction disturbances | 220 | 3,948·39 |  |  |  |
| Intracranial hemorrhage or cerebral infarction | 198 | 5,526·91 |  |  |  |
| Other diagnosis of the circulatory system | 108 | 3,623·26 |  |  |  |
| Circulatory Diseases Except AMI with cardiac catheterization | 107 | 9,438·46 |  |  |  |
| **Heart Transplant ^(2)^** | **106·35** ^(6)^ | **27,705·60** |  | **0·00012** | Martins-Melo et al. (2014)^19^, Almeida (2016)^20^, Nascimento et al. (2024)^21^ |
| **Surgical inpatient care for Digestive Chagas disease ^(3)^** | **198** | **12,341·32** | **1 (per year)** | **0·029** | IBGE (2020)^4^ |
| Infectious and parasitic diseases with surgery | 92 | 15,108·88 |  |  |  |
| Major Surgeries of the Large and Small Intestine | 71 | 10,183·01 |  |  |  |
| Surgeries of the Stomach, Esophagus, and Duodenum | 19 | 9,003·42 |  |  |  |
| Other surgeries of the digestive system | 16 | 9,969·12 |  |  |  |
| **Clinical inpatient care for Digestive Chagas disease ^(3)^** | **225** | **3,068·22** | **1·63 (per year)** | **0·169** | IBGE (2020)^4^ |
| Other infectious and parasitic diseases | 109 | 3,526·35 |  |  |  |
| Esophagitis, gastroenteritis, and other digestive diseases | 60 | 3,495·14 |  |  |  |
| Gastrointestinal obstruction | 56 | 1,719·10 |  |  |  |
| Note: (1) Hospitalizations with primary and secondary International Classification of Disease (ICD) as B57.2 or primary chapter I with secondary B57, based on DRG Brasil data from the base year 2024 ^22^. (2) Transplants provided by the public health system between 2015 and 2022 registered in the National Transplant System^23^, and estimated costs from Hospital Information System (SIH/DATASUS)^24^ in 2022. (3) Hospitalizations with primary and secondary ICD as B57.3, K23.1 and K93.1, DRG Brasil. (4) Number of patients who received care in the hospitals of the DRG-Brasil database. The number of patients was used as a weighting factor to calculate the average costs of each group of procedures. (5) This amount includes $33,970·00 (Purchasing Parity Power US dollars – PPP-USD) for the prosthesis (implantable cardiac defibrillator), sourced from a brazilian private health insurance company in 2024, and PPP-USD 9,458·40 for hospitalization, according to DRG Brasil data from 2024. (6) This value corresponds to the annual average number of transplants among Chagas disease patients with heart failure over the period 2015 to 2022. | | | | | |

Supplementary Table 8: Sensitivity analysis parameters

| **Parameter** | **Deterministic analysis** | **Sources** | **Tornado Diagram and Probabilistic analysis** | | **Sources** |
| --- | --- | --- | --- | --- | --- |
|  | **Value** |  | **Value** | **Distribution** |  |
| **Prevalence** | 1·02 | Dias et al. (2016)^16^ | - | - | - |
|  | 1·88 | Laporta et al. (2024)^25^ | - | - | - |
| **Distribution forms** |  | Nascimento et al. (2024)^26^ |  |  |  |
| *Overall studies* |  |  | - | - | - |
| Indeterminate | 42·6 |  | - | - | - |
| Cardiac | 37·1 |  | - | - | - |
| Digestive | 8·6 |  | - | - | - |
| Mixed | 11·7 |  | - | - | - |
| *Endemic countries* |  |  | - | - | - |
| Indeterminate | 36·8 |  | - | - | - |
| Cardiac | 39·2 |  | - | - | - |
| Digestive | 10·1 |  | - | - | - |
| Mixed | 13·9 |  | - | - | - |
| **Inpatient costs** |  | SIH/DATASUS (2022)^24^ | baseline - DRG (+/-20%) | Triangular | (+/-20% baseline parameter) |
| Surgical inpatient care - cardiac form | 3,761·95 |  | (13,713·72-20,570·59) |  |  |
| Clinical inpatient care - cardiac form | 1,428·08 |  | (4,621·70-6,932·55) |  |  |
| Surgical inpatient care - digestive form | 1,187·18 |  | (9,873·05-14,809·58) |  |  |
| Clinical inpatient care - digestive form | 325·91 |  | (2,454·58-3,681·87) |  |  |
| Heart transplant |  |  | (22,164·48-33,246·72) |  |  |
| **Absenteeism costs** ^(1)^ |  |  | baseline - GDP (+/-20%) | Triangular | (+/-20% baseline parameter) |
|  | 69·57 | IBGE (2023)^27^ | (72·86-109·30) |  |  |
| **Transition probabilities** |  |  |  |  |  |
| Cardiac to death | - | - | baseline (95% CI)  (0·063-0·101) | Uniform | Chadalawada et al. (2021)^11^ |
| Digestive to death | - | - | baseline (+/-20%)  (0.00052-0.00078) | Uniform | (+/-20% baseline parameter) |
| Mixed to death | - | - | baseline (95% CI)  (0·063-0·101) | Uniform | Chadalawada et al. (2021)^11^ |
| Indeterminate to cardiac | - | - | baseline (95% CI)  (0·012-0·043) | Uniform | Chadalawada et al. (2020)^6^ |
| Indeterminate to digestive ^(2)^ | - | - | baseline (+/-20%)  (0·0088-0·0132) | Uniform | (+/-20% baseline parameter) |
| Indeterminate to cure | - | - | baseline (r=8; n=100) | Beta | Ferreira et al. (1990)^28^ |
| Cardiac to mixed | - | - | baseline (+/-20%)  (0·0088-0·0132) | Uniform | (+/-20% baseline parameter) |
| Digestive to mixed | - | - | baseline (95% CI)  (0·012-0·043) | Uniform | Chadalawada et al. (2020)^6^ |

Note: (1) Absenteeism costs were estimated using 2023 data from the PNADC (Continuous National Household Sample Survey) Absenteeism costs were not included in the tornado diagram analysis. (2) No interval for the transition probabilities from indeterminate to digestive was found in literature. Therefore, the limits were defined by varying the baseline value by +/- 20%.

References

1 Husereau D, Drummond M, Augustovski F, *et al.* Consolidated Health Economic Evaluation Reporting Standards 2022 (CHEERS 2022) statement: updated reporting guidance for health economic evaluations. *MDM Policy Pract* 2022; **7**: 23814683211061097.

2 Lee BY, Bacon KM, Bottazzi ME, Hotez PJ. Global economic burden of Chagas disease: a computational simulation model. *Lancet Infect Dis* 2013; **13**: 342–8.

3 Cardoso CS, Sabino EC, Oliveira CDL, *et al.* Longitudinal study of patients with chronic Chagas cardiomyopathy in Brazil (SaMi-Trop project): a cohort profile. *BMJ Open* 2016; **6**: e011181.

4 Instituto Brasileiro de Geografia e Estatística (IBGE). National Health Survey 2019 – Pesquisa Nacional de Saúde – PNS. 2020. https://www.ibge.gov.br/estatisticas/sociais/saude/9160-pesquisa-nacional-de-saude.html.

5 GBD 2019 Diseases and Injuries Collaborators, Lim SS, Abbafati C, *et al.* Global burden of 369 diseases and injuries in 204 countries and territories, 1990–2019: a systematic analysis for the Global Burden of Disease Study 2019. *The Lancet* 2020; **396**: 1204–22.

6 Chadalawada S, Sillau S, Archuleta S, *et al.* Risk of Chronic Cardiomyopathy Among Patients With the Acute Phase or Indeterminate Form of Chagas Disease: A Systematic Review and Meta-analysis. *JAMA Netw Open* 2020; **3**: e2015072.

7 Castro C, Macêdo V, Rezende JM, Prata A. Estudo radiológico longitudinal do esôfago, em área endêmica de doença de Chagas, em um período de 13 anos. *Rev Soc Bras Med Trop* 1994; **27**: 227–33.

8 Instituto Brasileiro de Geografia e Estatística (IBGE). Tábua completa de mortalidade para o Brasil. IBGE Bibl. 2011. https://biblioteca.ibge.gov.br/index.php/biblioteca-catalogo?view=detalhes&id=73097 (accessed April 22, 2024).

9 Andrade ALSSD, Zicker F, De Oliveira RM, *et al.* Randomised trial of efficacy of benznidazole in treatment of early Trypanosoma cruzi infection. *The Lancet* 1996; **348**: 1407–13.

10 Ferreira HDO. Tratamento da forma indeterminada da doença de Chagas com nifurtimox e benzonidazol. *Rev Soc Bras Med Trop* 1990; **23**: 209–11.

11 Chadalawada S, Rassi A, Samara O, *et al.* Mortality risk in chronic Chagas cardiomyopathy: a systematic review and meta‐analysis. *ESC Heart Fail* 2021; **8**: 5466–81.

12 Wilson LS, Strosberg AM, Barrio K. Cost-effectiveness of Chagas disease interventions in Latin America and the Caribbean: Markov models. 2005; published online Nov 1. DOI:10.4269/ajtmh.2005.73.901.

13 Organización Panamericana de la Salud. Guía para el diagnóstico y el tratamiento de la enfermedad de Chagas. 2018. https://iris.paho.org/handle/10665.2/49653.

14 Echeverría LE, Marcus R, Novick G, *et al.* WHF IASC Roadmap on Chagas Disease. *Glob Heart* 2020; **15**: 26.

15 Comissão nacional de incorporação de tecnologias ao SUS (CONITEC), Ministério da Saúde- Brasil. Protocolo Clínico e Diretrizes Terapêuticas para Doença de Chagas: relatório de Recomendação — Ministério da Saúde. https://www.gov.br/saude/pt-br/centrais-de-conteudo/publicacoes/svsa/doenca-de-chagas/protocolo-clinico-e-diretrizes-terapeuticas-para-doenca-de-chagas-_-relatorio-de-recomendacao.pdf/view (accessed Nov 26, 2024).

16 Dias JCP, Novaes Ramos A, Dias Gontijo E, *et al.* II Consenso Brasileiro em Doença de Chagas, 2015. *Epidemiol E Serviços Saúde* 2016; **25**: 1–10.

17 Fundação Oswaldo Cruz (FIOCRUZ). Internações da Saúde Suplementar - Procedimentos e eventos em saúde. https://tabnet.fiocruz.br/dhx.exe?tiss/fat_internacao_procedim.DEF (accessed June 30, 2024).

18 Câmara de Regulação do Mercado de Medicamentos (CMED). Listas de preços de medicamentos. 2024. https://www.gov.br/anvisa/pt-br/assuntos/medicamentos/cmed/precos/capa-listas-de-precos (accessed July 25, 2024).

19 Martins-Melo FR, Ramos AN, Alencar CH, Heukelbach J. Prevalence of Chagas disease in Brazil: A systematic review and meta-analysis. *Acta Trop* 2014; **130**: 167–74.

20 Almeida DR. Transplante cardíaco na Doença de Chagas. *Rev Soc Cardiol Estado Säo Paulo* 2016; : 266–71.

21 Nascimento BR, Naback ADN, Santos BMP, *et al.* Prevalence of clinical forms of Chagas disease: a systematic review and meta-analysis – data from the RAISE study. *Lancet Reg Health - Am* 2024; **30**: 100681.

22 DRG Brasil. DRG Brasil. Versão 15.4.12. [s.l.]. https://sigclinic.sigquali.com.br/qualidade/inicial.do (accessed Feb 1, 2024).

23 Ministério da Saúde do Brasil. Sistema Nacional de Transplantes. Sist. Nac. Transpl. https://www.gov.br/saude/pt-br/composicao/saes/snt/transplantes (accessed June 7, 2024).

24 Ministério da Saúde do Brasil. SIH- Sistema de Internações Hospitalares. 2022. https://datasus.saude.gov.br/ (accessed Oct 2, 2023).

25 Laporta GZ, Lima MM, Maia Da Costa V, *et al.* Estimativa de prevalência de doença de Chagas crônica nos municípios brasileiros. *Rev Panam Salud Pública* 2024; **48**: 1.

26 Nascimento BR, Naback ADN, Santos BMP, *et al.* Prevalence of clinical forms of Chagas disease: a systematic review and meta-analysis – data from the RAISE study. *Lancet Reg Health - Am* 2024; **30**: 100681.

27 Instituto Brasileiro de Geografia e Estatística (IBGE). PNAD Contínua - Pesquisa Nacional por Amostra de Domicílios Contínua 2023. https://www.ibge.gov.br/estatisticas/sociais/trabalho/17270-pnad-continua.html?edicao=39587 (accessed Nov 6, 2024).

28 Ferreira HDO. Tratamento da forma indeterminada da doença de Chagas com nifurtimox e benzonidazol. *Rev Soc Bras Med Trop* 1990; **23**: 209–11.
