# Supplementary material for: Economic burden of Chagas disease in Brazil: a nationwide cost-of-illness study
Source: Lancet Reg Health Am. 2025 Aug 8;50:101202. doi: 10.1016/j.lana.2025.101202 (PMC12356041; doi:10.1016/j.lana.2025.101202)
Supplement: Abstract-portuguese [file mmc2.docx]

**Editorial disclaimer**

The translation of the Summary was submitted by the authors, and we reproduce it as supplied. It has not been peer reviewed. Our editorial processes have only been applied to the original version in English, which should serve as a reference for this manuscript.

**Título: A carga econômica da doença de Chagas no Brasil: um estudo nacional de custo da doença**

**Resumo**

**Contexto:** A doença de Chagas continua sendo um problema de saúde pública com impacto financeiro substancial no sistema de saúde dos países latino-americanos. Apesar de sua elevada carga econômica, pesquisas que quantificam os custos diretos e indiretos são limitadas, particularmente no Brasil. Este estudo estima a carga econômica da doença de Chagas crônica no Brasil, como parte do projeto mais amplo, “A Carga da Doença de Chagas no Mundo Contemporâneo: O estudo RAISE”.

**Método:** Foi utilizado um modelo de Markov para estimar a carga econômica da doença de Chagas crônica a partir de uma perspectiva social, considerando seis estados de saúde mutuamente excludentes: quatro formas clínicas (indeterminada, cardíaca, digestiva e mista) e dois estados absorventes (morte e cura). Este modelo foi estimado por meio de uma microssimulação, com ciclos anuais, considerando uma coorte hipotética de 10.000 pacientes, cada um repetido 1.000 vezes para se obter a média. Os dados de custos foram coletados em Reais (R$) e convertidos para dólares americanos de paridade de poder de compra de 2024 (PPP-USD). Foram estimados custos médicos diretos e perdas de produtividade devido ao absenteísmo.

**Resultados:** A carga econômica anual da doença de Chagas crônica no Brasil foi estimada em $11,44 bilhões, representando 0,23% do produto interno bruto, com um custo ao longo do ciclo de vida de $45.034 por paciente. Os custos médicos diretos ao longo da vida representam cerca de 72% da carga econômica total, enquanto os custos indiretos, 28%. Os custos médicos diretos anuais representam cerca de 11% do orçamento do Ministério da Saúde.

**Interpretação:** A elevada carga econômica da doença de Chagas crônica destaca a necessidade de políticas eficazes de saúde pública e alocação de recursos no sistema de saúde brasileiro. Estimar os custos considerando o modelo de cobertura universal de saúde no Brasil pode guiar políticas e intervenções visando a redução do impacto da doença de Chagas.
